# The pressure to communicate efficiently continues to shape language use later in life

**DOI:** 10.1038/s41598-020-64475-6

**Published:** 2020-05-19

**Authors:** Madeleine Long, Hannah Rohde, Paula Rubio-Fernandez

**Affiliations:** 1 Department of Philosophy, University of Oslo, Oslo, Norway; 2 Department of Linguistics and English Language, University of Edinburgh, Edinburgh, UK; 3 Department of Brain and Cognitive Sciences, MIT, Cambridge, USA

**Keywords:** Cognitive ageing, Human behaviour

## Abstract

Language use is shaped by a pressure to communicate efficiently, yet the tendency towards redundancy is said to increase in older age. The longstanding assumption is that saying more than is necessary is inefficient and may be driven by age-related decline in inhibition (i.e. the ability to filter out irrelevant information). However, recent work proposes an alternative account of efficiency: In certain contexts, redundancy facilitates communication (e.g., when the colour or size of an object is perceptually salient and its mention aids the listener’s search). A critical question follows: Are older adults indiscriminately redundant, or do they modulate their use of redundant information to facilitate communication? We tested efficiency and cognitive capacities in 200 adults aged 19–82. Irrespective of age, adults with better attention switching skills were redundant in efficient ways, demonstrating that the pressure to communicate efficiently continues to shape language use later in life.

## Introduction

Human communication is shaped by an underlying pressure for efficiency^1–3^. Recent studies have shown that efficiency pressures apply across languages, shaping the way speakers rely on context to establish the meaning of a message (what is known as *pragmatics*)^4,5^. From a developmental perspective, an important question remains as to whether speakers are subject to efficiency pressures across the human lifespan. Most adult speakers respond to the pressure to be efficient by staying on topic and avoiding excess wordiness, a demanding process which may involve executive functions such as inhibition (i.e. the ability to filter out irrelevant information). While younger adults are generally considered succinct speakers^6^, older adults are often viewed as unnecessarily verbose^7^, perhaps due to deficits in inhibitory control^8^. However, the analysis of efficiency has largely neglected the idea that in certain contexts, redundant information can facilitate communication^9,10^. Here we test whether adults of all ages are redundant in contexts where it aids listener comprehension, and what cognitive skills are necessary to be efficient in this way. We propose that efficiency requires monitoring for contextual cues that licence the use of extra information and that individual differences in attention switching may underlie this ability to adjust communicative strategies in context-dependent ways^11^.

### Ageing and redundancy

Redundancy in ageing is a topic familiar to many of us. Most of us have had the experience of conversing with an elderly person only to find them veering off-topic or being overly descriptive. A prominent line of research has studied this phenomenon in an effort to understand the extent to which older adults are less efficient communicators. However, thirty years of research has provided mixed results. In an influential study^12^, James *et al*. found that older adults’ off-topic verbosity was confined to autobiographical stories and did not extend to other contexts such as picture descriptions. The authors explain these results through the *pragmatic change account*, which posits that when older adults discuss life events, they shift their conversational goals from the concise exchange of information to an emphasis on personal narratives. In line with this account, a more recent study found that age-related differences in off-topic verbosity only emerged in contexts where younger and older adults’ conversational goals diverged^13^. Specifically, when discussing procedural life events (such as daily routines), younger adults expressed a preference for succinctness, while older adults expressed a preference for expressiveness, which may explain why older adults displayed greater off-topic verbosity in that context. On the other hand, when discussing episodic life events (such as a favourite vacation), both younger and older adults preferred to be expressive and no differences in off-topic verbosity were found. A competing account, the *inhibitory deficit hypothesis*, challenges these findings, providing an alternative explanation for older adults’ behaviour: Off-topic verbosity is not confined to specific contexts (e.g., autobiographical ones), but rather extends to all aspects of language production as it is caused by age-related deficits in the ability to inhibit irrelevant information^14–16^. Supporting this hypothesis, Arbuckle *et al*.^17^ found that older adults with high off-topic verbosity were more likely to use redundant descriptions in a task that did not involve personal narratives, but that tested referential communication (i.e. the way in which we refer to people or things).

These conflicting findings may be due to the fact that efficiency in referential communication is often measured only on the basis of *informational value*. That means that descriptive information (such as colour) is only considered valuable if it is used to distinguish the intended referent from other entities of the same kind, pre-empting an ambiguity. Imagine that you are at school pick up and want to point out your child to a new acquaintance: You might say “my child is the one wearing a blue shirt”. However, this description is only useful if the other children are wearing different coloured shirts. If your child were standing next to a football team whose shirts also happened to be blue, then mentioning the colour of your child’s shirt would have no informational value as it does not distinguish your child from the other children.

While a description’s informational value clearly contributes to its degree of efficiency, informational value alone fails to account for perceptual factors such as visual salience or discriminability^9,10^. Imagine this time your child is surrounded by children wearing football team jackets. It would be much easier for your acquaintance to identify your child from the description “my child is the one wearing a blue shirt” if the football jackets were white, rather than blue. Regardless of the colour of the jackets, the inclusion of the colour modifier “blue” would be redundant since the property “wearing a shirt” would be sufficient to identify your child in the crowd. However, when colour is distinctive (i.e. when there is only one blue item of clothing in the scene), using colour redundantly may facilitate the listener’s visual search because of its *discriminatory value*. Therefore, instead of focusing solely on the informational value of a description, theories of efficiency should also consider its discriminatory value in the visual context.

**Figure 1:**
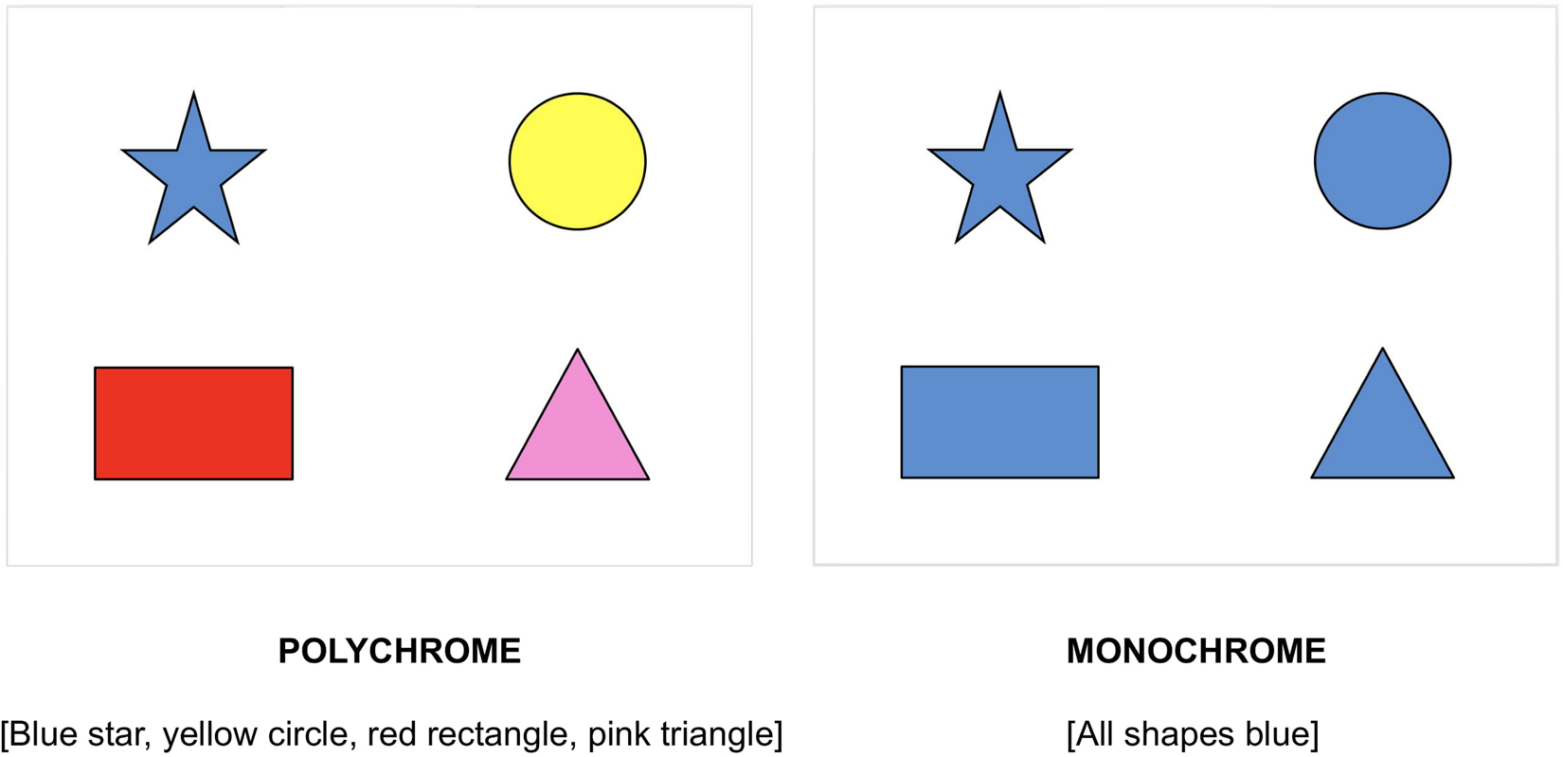
Sample polychrome and monochrome displays.

Recent work addresses these issues, offering a more nuanced definition of efficiency: *A description is efficient not only when it is short, but also if it facilitates the listener’s identification of the target referent*^9,10^. Supporting this view are eye-tracking results which show that in contexts where a target’s colour is distinctive, the inclusion of redundant colour adjectives (RCAs) aid listeners’ identification of the target. For example, in the polychrome display in Fig. 1, a redundant description such as ‘the blue star’ speeds object identification relative to the bare description ‘the star’, whereas the same RCA in the monochrome display delays the visual search as it creates a temporary ambiguity until the target shape is specified^18^ (see also^19–25^).

Complementing this finding, language production data show that young adults who were shown polychrome displays often produced descriptions with RCAs whereas younger adults who were shown monochrome displays rarely produced RCAs, demonstrating that young adults are sensitive to this kind of context-dependent discriminatory efficiency^9,10,26,27^. Nevertheless, there is a critical limitation to this work: in order to avoid carry-over effects, participants were only shown monochrome or polychrome displays, but not a combination of the two, such as one might expect to encounter in the changing contexts of more naturalistic discourse. In the current study, we address this limitation by investigating participants’ sensitivity to a change in display type and the associated pressure to change modification strategies. In addition to this, we extend previous work by investigating both older and younger adults’ use of RCAs. Given older adults’ propensity for redundancy, we were interested in whether they are still sensitive to efficiency pressures, or whether they no longer appreciate when redundancy has discriminatory value.

### Ageing and cognitive control

Though it is well-documented that older adults are more verbose than younger adults, the source of this redundancy is less clear. Research from the past fifty years has revealed a complex and multifaceted picture of the impact of cognitive ageing on linguistic abilities^28^. On the one hand, as individuals grow older, they become more susceptible to decline in domain-general cognitive functions, which studies suggest can lead to less coherent speech^29,30^ and a reduced ability to tailor their speech to their listener’s needs^31–33^. Indeed, studies have found that Theory of Mind  abilities may decline with age, reflecting a decrease in sensitivity to an interlocutor’s perspective, including what information should or should not be shared based on the interlocutor’s knowledge and beliefs^34–36^. On the other hand, the ageing brain is capable of remarkable plasticity, which may make older adults (with a lifetime of language experience) better equipped to adopt effective production strategies based on available cognitive resources^37–40^. Recent work suggests that individual differences in response to ageing may account for divergent linguistic outcomes: While some older adults are indistinguishable from younger adults in perspective-taking^11^, fluency^41,42^, and ambiguity avoidance^43^, others display marked decline in the same areas^6,33,44^.

Much of the cognitive ageing literature has focused on whether or not older adults are resilient in the face of cognitive decline. However, less consideration has been given to the possibility that different cognitive strategies may be employed at different stages of life. That is, rather than assuming older adults’ linguistic behaviour is related to cognitive deficits or cognitive resilience, a better approach may be to explore which modes of cognitive control are preferred at different ages. According to Braver’s dual-mechanisms of control framework^45^, an individual’s ability to regulate thoughts and actions (such as utterance planning) requires a complex balance between proactive and reactive modes of cognitive control. The proactive mode involves prioritizing the maintenance of internal goals while preventing interference from distracting information (thus can be viewed as an index of inhibitory control); The reactive mode involves monitoring background information and flexibly updating goal representations in response to contextual changes (thus can be viewed as an index of attention switching)^11,46^.

Proactive control relies on the anticipation and prevention of interference (i.e. top-down processing), whereas reactive control relies upon the detection and resolution of interference after its onset (i.e. bottom-up processing). There are costs and benefits associated with each mode of control, thus depending on the communicative scenario, a speaker may favour one mode of control over the other. The advantage of a proactive mode of control is that it allows a speaker to optimize preparation of an utterance while minimizing interference by *inhibiting* distracting information in the environment. The disadvantage is that this mode of control requires continuous goal maintenance, reducing one’s capacity to integrate new and potentially relevant contextual information and update one’s utterance when appropriate. The reactive mode of control, on the other hand, has the advantage of being more computationally efficient: A speaker’s goal representation for an utterance is only activated (or re-accessed) when necessary, thus freeing up cognitive resources. The disadvantage is that there is less reliance on sustaining one’s goal representation for an utterance and greater dependence on *switching attention* to relevant contextual cues in the environment (such as changes in the salience or discriminability of an object in a scene) in order to trigger an appropriate response.

To illustrate how these modes of control are relevant for redundancy, let’s revisit the school pick up scenario. If your goal as a speaker is to use minimal expressions, and you prefer a proactive mode of control, then you would bias attention to that goal (at the cost of inhibiting potentially relevant discriminatory information such as the colour of your child’s shirt) in order to achieve a minimal expression. This type of “early selection” mechanism involving strong interference control would result in an efficient description in a scenario where your child is wearing a blue shirt and is standing near the baseball team, who are all wearing blue jackets. However, school pick ups can be quite chaotic as children move around, so if your child went over to talk to the basketball team and they were wearing orange jackets, then mentioning the colour of your child’s shirt would actually facilitate the listener’s visual search. Thus, in situations where the visual context varies, it would be useful to employ a reactive mode of control which focuses less on goal-maintenance (i.e. the use of minimal or full expressions across contexts), and more on flexibility (i.e. the readiness to disengage inhibition and refocus attention to appropriate contextual information) as a “late correction” mechanism. In this way, a speaker’s goal may change from giving minimal descriptions to giving full descriptions in situations where it would aid a listener’s visual search. As such, a reactive mode of control involving the ability to shift attention to changing contextual information would allow speakers to appropriately update redundant modification strategies according to the salience or discriminability of an object in a scene.

Given that a redundant description should only be used in contexts where it is helpful for the listener, we propose that a reactive mode of control involving attention switching would be particularly well-suited for the use of facilitatory redundancy. We also propose that attention switching may be the preferred mode of control for both younger and older adults in situations where the referential context changes. Supporting this hypothesis is recent work showing that attention switching predicts behaviour in an interactive task that requires taking the listener’s perspective in different contexts^11^. Furthermore, recent behavioural and neural research has found an increase in reactive control with ageing^47^, which may reflect a strategic decision on the part of older adults since attention switching relies on a bottom-up ‘late correction’ mechanism which is computationally efficient.

## The current study

In the current study, we investigated the nature and source of redundancy across the adult lifespan, treating participant age (19–82) as a continuous variable. To address these issues, we administered a referential communication task in which participants were shown displays (as in Fig. 1) and asked to indicate which shape was the target in each display, so that the experimenter could find it. The nature of redundancy was assessed by manipulating two aspects of the visual context:

- ***Display Type***: Each participant was presented with monochrome and polychrome displays. In monochrome displays, mentioning the color of the target would be inefficient as it would make the speaker’s utterance longer in addition to creating a temporary ambiguity for the listener, hindering the listener’s visual search for no communicative benefit. In polychrome displays, on the other hand, a speaker can be efficient in one of two ways: by either mentioning the color of the target, making the utterance longer but speeding up the listener’s visual search, or by using a minimal expression (i.e. mentioning only the shape of the target) making the utterance shorter but not going out of one’s way to aid the listener’s visual search. A speaker’s choice of strategy (i.e. being minimal or aiding the listener’s search) likely depends on other aspects of the discourse, namely the order in which a speaker encounters these display types and whether they choose to switch strategies to be more efficient and avoid inappropriate redundancy for the monochrome display type.
- ***Order***: Participants were randomly assigned to one of two trial-block orders: polychrome-monochrome order or monochrome-polychrome. This manipulation allowed us to vary the efficiency pressure to switch RCA strategies: Those presented with polychrome trials in block 1 are under greater pressure to switch out of colour modification as colour becomes inefficient in block 2, hindering the listener’s visual search for no communicative benefit (e.g., “blue star” in a monochrome display). Those presented with monochrome trials in block 1, on the other hand, are under less pressure to switch into color modification in block 2 since a minimal expression would not hinder the listener’s visual search (e.g., “star” in a polychrome display).

In addition to the contextual manipulations above, we added a third, higher-level task version manipulation (i.e. the presence or absence of multi-colored fillers) to assess general communicative strategies (i.e. whether speakers of different ages are more or less inclined towards redundancy by more frequently opting for RCAs over bare nouns). Research has shown that while speakers do not default entirely to a strategy of brevity or redundancy, they tend to refer to objects in consistent ways^48^, and that depending on the situation, younger and older adults differ in their communicative goals^13^ with younger adults prioritising brevity more often than older adults. However, it is unclear whether younger adults would prioritise brevity in the version with fillers as the likelihood of encountering a multi-colored display greatly increases. Thus, in addition to testing the nature of redundancy, we were also interested in testing whether a higher-level task factor would reveal age-related differences in participants’ general communicative strategies and whether sensitivity to this high-level factor would influence participants’ overall strategy. To test this, we varied the task version by the presence or absence of multi-coloured fillers:

- ***Task Version:*** Participants were randomly assigned to one of two versions of the task: critical trials only or critical trials plus 40 fillers interspersed. The critical trials were the same in both versions of the task, including the order in which they were presented. In the version with fillers, the target was a unique colour in half of the filler trials, and in the other half, the target was the same colour as another shape in the display. This manipulation allowed us to investigate age-related differences in general communicative strategies^13^ and in sensitivity to the proportion of multi-colored displays presented during the task.

To assess the source of redundancy, we separately tested participants’ inhibitory control and attention switching skills, along with a baseline measure of sustained attention. In addition, we measured participants’ disfluency (e.g., filled pauses, self-repairs, and elongated sounds). Disfluency can be viewed as an index of difficulty with utterance planning^44,49^, which can shed light on the source of redundancy: If participants rely on attention switching, those with better switching skills will rapidly respond to contextual changes during utterance planning, revealing less disfluency. Below we outline the main predictions for our hypotheses. Note that while we tested both attention switching and inhibition, our predictions are based on the view that in situations where the referential context changes, attention switching may be the preferred mode of control for both younger and older adults. We tested inhibitory control alongside attention switching only because it is a frequent measure of executive control in ageing research^28^, but we did not have grounds to predict that it would affect participants’ RCA strategies.

Regarding the nature of redundancy over the lifespan, two predictions can be made:

(i) If sensitivity to efficiency pressures does not decline with age, then we would expect an effect of context but no additional interaction with age.
(ii) If an increase in age decreases sensitivity to efficiency pressures, then we would expect an interaction of context and age showing that as age increases sensitivity to the use of RCAs in appropriate contexts decreases.

Regarding the source of redundancy over the lifespan, two predictions can be made:

(iii) If participants of all ages are redundant in efficient ways through the use of a context-driven reactive mode of cognitive control, then we would expect to find that age does not interact with switching and context to predict efficiency. Instead, better switchers of all ages would use RCAs in context-sensitive ways.
(iv) If an increase in age increases the likelihood that participants are indiscriminately redundant because they do not rely on a context-driven reactive mode of control, then we would expect to see that switching abilities become less predictive of context sensitivity as age increases.

Regarding general communicative strategies over the lifespan, two predictions can be made:

(v) If younger and older adults differ in their general communicative strategies (with younger adults favouring a strategy of brevity, as indicated in previous research), we would expect older adults to use more RCAs than younger adults in the version of the task without fillers. However, this difference would likely be less pronounced (or non-existent) in the version of the task with fillers where the likelihood of encountering a multi-colored display greatly increases and thus maintaining a strategy of brevity would become less relevant for younger adults.
(vi) If younger and older adults do not differ in their general communicative strategies, we would expect to find no age-related difference in RCA rates across either version of the task.

In addition to our main predictions, additional predictions can be made regarding the source of redundancy based on participants’ rate of disfluency:

(vii) If switching abilities underlie referential efficiency across the lifespan, then we would expect disfluency to decrease with better switching skills, as a better ability to shift between including and omitting RCAs in this task should result in less difficulty with utterance planning.
(viii) If participants rely less on switching skills for referential efficiency as age increases, then we would expect to see that switching become less predictive of performance as age increases.

## Methods

### Participants

**Figure 2:**
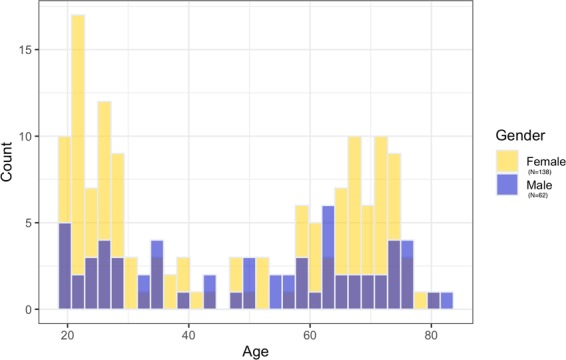
Histogram of age and gender within the sample.

A total of 209 participants were recruited from the University of Edinburgh volunteer panel, the university careers services website, and local community groups. The study was approved by the University of Edinburgh Linguistics and English Language ethics committee and written informed consent was obtained from each participant prior to testing. All research was performed in accordance with the relevant guidelines and regulations. There were 9 participants excluded from the analysis: 6 non-native English speakers, 1 participant with hearing issues, 1 with abnormally low attentional scores, and 1 due to a technical malfunction. We thus report data from 200 native English speakers aged 19–82 with normal colour vision and hearing (see Fig. 2 for distribution of age and gender within the sample).

### Materials and Procedure

#### Referential communication task

This task was adapted from Rubio-Fernandez^10^. Twenty displays of 4 different shapes were designed such that each shape appeared in one of the quadrants (see Fig. 1). A total of 10 possible shapes (arrow, circle, cylinder, heart, oval, pentagon, rectangle, square star and triangle) and 9 possible colours (blue, brown, grey, green, orange, pink, purple, red and yellow) were randomly combined to create each of the displays. Critical trials consisted of polychrome displays (10 trials) in which all shapes were a different colour, and monochrome displays (10 trials) in which all shapes were the same colour. In the version with fillers, filler trials (n = 40) were a hybrid of polychrome and monochrome displays such that there were 4 shapes of 3 colours (i.e. one of the colours was repeated). During the task, participants were asked to indicate which shape was the target in each display, so that the experimenter could click on it. Crucially, the four shapes presented in the displays were always different, so a bare shape description (e.g., “the star”) would provide sufficient information to identify the target. Mentioning the colour of the shape would be redundant but efficient in polychrome displays, and redundant and inefficient in monochrome displays. The shape alone is not inefficient, though it may lack the discriminatory power of color for visual search.

Participants’ responses were audio recorded for transcription and coding purposes. Prior to commencing the task, participants were instructed to sit behind the experimenter and were given a print-out of blank grids, one for each trial, with the trial number on the top of the page. On each grid, an X marked the quadrant where the target shape was located on the experimenter’s computer screen. The position of the target changed with each trial. Participants were told to avoid using coordinates (e.g., ‘top left’) when referring to the target.

#### Test of Everyday Attention

Participants’ attentional skills were measured via the Test of Everyday Attention (TEA)^50^, a well-established clinical test based on Posner and Peterson’s multi-system attentional model^51^. The test offers a fine-grained method of assessing an individual’s cognitive resources by separating attention into theoretically distinct factors through the use of three auditory subtests: switching, inhibition, and sustained attention. The subtests are closely-related, thus eliminating the influence of task differences on cognitive performance, and the subtests are argued to have high ecological validity as they are structured around a plausible real-life scenario^52^. The TEA was originally designed to monitor the effects of neuro-rehabilitation in clinical populations, thus is sensitive enough to detect subtle differences in attention across a wide range of ages. Moreover, it has been standardized through a normative sample of healthy adults aged 18–80^53^, making it particularly suitable for the age range in this study. Crucially, the TEA has been successfully applied to test the relationship between attentional skills and a wide range of linguistic abilities across the adult lifespan, from perspective-taking^11^ and pronominal use^46^ to bilingualism^54,55^ and language learning^56^ and thus was adopted for the purpose of this study.

For each of the three auditory subtests (referred to as the elevator tasks), participants are asked to envision that they have entered an elevator on the ground floor. The floor light indicator does not work, so in order to know which floor they are on they must count the tones they hear. After each trial they are asked which floor they are on. Performance on each subtest is measured as the percentage of trials with correct responses (0–100).

#### *Elevator Task* (sustained attention, n = 7 trials)

Participants are presented with tones of the same pitch at irregular intervals and must keep track of the count. This task is not computationally difficult, but requires that participants pay attention. Healthy individuals are expected to perform near ceiling, therefore this was used as a baseline measure.

#### *Elevator Task with Distraction* (inhibition, n = 10 trials)

Participants are presented with low and high tones. They must selectively attend to and count the low tones while ignoring interspersed high tones. Performing well requires inhibiting the high tones while counting the low tones or selectively attending to the low tones only.

#### *Elevator Task with Reversal* (attention switching, n = 10 trials)

Participants are presented with low, medium, and high tones in random order. They must count medium tones only. Low tones indicate the elevator will move down with the subsequent medium tones, while high tones indicate the elevator will move up with subsequent medium tones. Performing well requires inhibiting low and high tones from the count while efficiently disengaging inhibition and refocusing attention upon hearing a middle tone.

## Results

### Results from initial assessment

**Figure 3:**
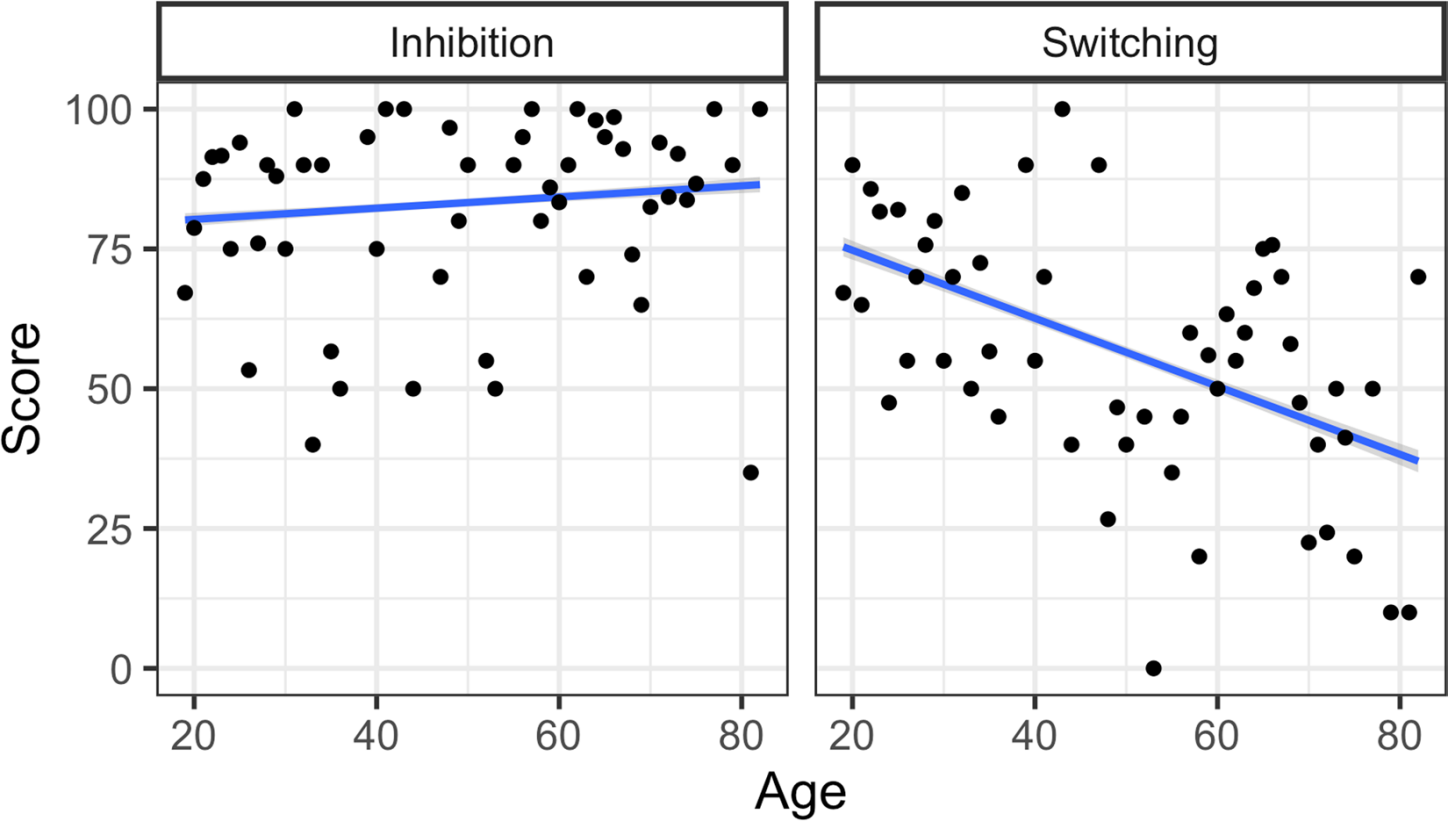
Inhibition and switching scores by age. Regression lines reflect the best fit of data, points reflect mean score for each age tested.

In order to select which attentional measures to include in the main analysis, we performed an initial assessment. As expected, participants performed at ceiling on the baseline measure of sustained attention (mean score=99.21), thus this will not be considered further. Next, we used regression analyses to separately examine the role of switching and inhibition in modulating referential decisions. The alpha level for all reported tests was set to 0.05 and all analyses were run using R statistical software^57^. The results confirmed our predictions, revealing that switching predicted RCA rates (*β* = 0.002, SE = 0.001, *t* = 3.068, *p* = 0.003) but that inhibition did not (*β* = −0.001, SE = 0.001, *t* = −0.259, *p* = 0.796). We also tested whether participant age predicted switching and inhibition performance and found that age predicted switching (*β* = −0.608, SE = 0.112, *t* = −5.431, *p* < 0.001), but not inhibition (*β* = 0.100, SE = 0.077, *t* = 1.298, *p* = 0.196). An examination of the raw data showed that despite the large sample size (n = 200) and wide range of ages (19–82), inhibition scores did not vary to a large degree. Moreover, participants’ inhibition performance showed no detectable decline as age increased (see Fig. 3), supporting results from a recent meta-analysis^58^. Instead, the same participants demonstrated greater variability on switching performance. Based on these initial findings, which revealed no role for inhibition in referential choice or age-related decline, we did not include inhibition in our main analyses. (Note that, as predicted, the inclusion of inhibition in the main analysis yielded no significant main effect of inhibition or interactions with inhibition.) Instead, we focused on the role of switching in predicting referential efficiency across the lifespan.

### Statistical analyses

#### Modelling decisions

Given that the inclusion of all individual difference measures would have risked an overspecified model (with eight factors and their interactions) that would  yield problems with convergence, we targeted for inclusion the manipulated factors (Display Type, Order, Task Version) and the ones implicated in our primary predictions (Age, Switching), and we excluded those for which we didn’t have specific predictions (Gender) and those that did not show a link to referential behaviour in this task (Sustained Attention, which was at ceiling, and Inhibition, which did not vary with age or with RCA rates).

#### Main analysis: rate of RCAs

Using logistic mixed effects regression, we modelled the binary outcome variable of presence/absence of RCAs (bare noun = 0, RCA = 1) with Age, Switching, Display Type, Order, and Task Version as fixed effects. Age and Switching were entered as scaled continuous predictors while deviation coding was used for Display Type (Mono = −0.5, Poly = 0.5), Order (Mono-Poly = −0.5, Poly-Mono = 0.5), and Task Version (With Fillers = −0.5, Without Fillers = 0.5). By-participant random intercepts and slopes were used for Display Type and by-item random intercepts and slopes were used for Age, Switching, Display Type, Order, and Task Version and their interactions (i.e. the maximal random effect structure for participants and items^59^). Note that the model converged after removing correlations between random effects.

#### Additional analysis: rate of disfluency

Using logistic mixed effects regression, we modelled the binary outcome variable of presence/absence of disfluent speech (fluent = 0, disfluent = 1) with the same fixed effects as in the main analysis: Age, Switching, Display Type, Order, and Task Version. Again, by-participant random intercepts and slopes were used for Display Type and by-item random intercepts and slopes were used for Age, Switching, Display Type, Order, and Task Version and their interactions (i.e. the maximal random effect structure for participants and items^59^). Note that the model converged after removing correlations between random effects.

### Main results: rate of RCAs

**Figure 4:**
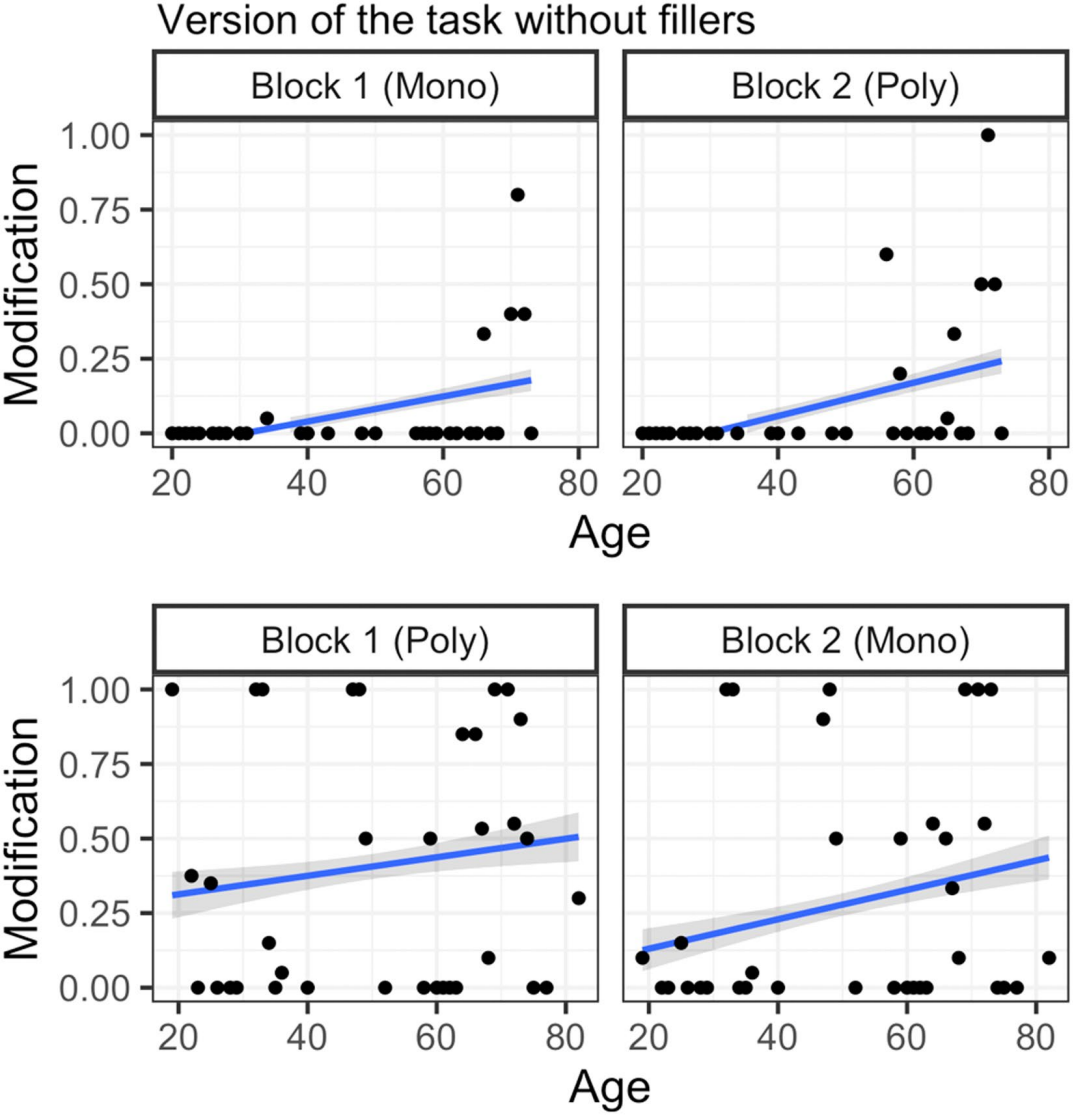
Rate of RCAs by age and order on the version of the task without fillers. Regression lines reflect the best fit of data, points reflect mean rate of RCAs for each age tested.

**Figure 5:**
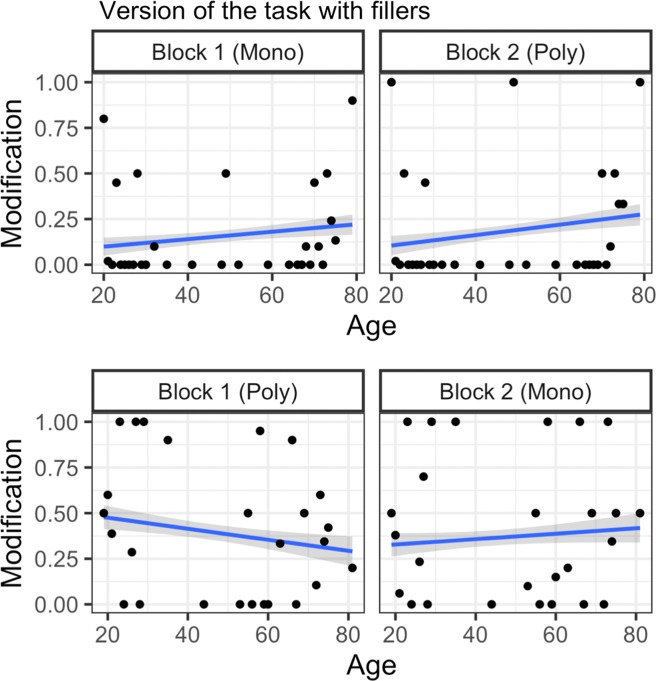
Rate of RCAs by age and order on the version of the task with fillers. Regression lines reflect the best fit of data, points reflect mean rate of RCAs for each age tested.

Results revealed clear evidence for the nature and source of redundancy over the lifespan. For a general overview of the data, see descriptive statistics in Tables 1 and 2 for rate of RCAs divided by younger and older adults through a median age split. Figures 4 and 5 show a more fine-grained representation of the data by illustrating participants’ RCA rate across all ages.

**Table 1:** Rate of RCAs for younger adults on the referential communication task.

Version of the task without fillers						
	Monochrome-Polychrome			Polychrome-Monochrome		
	Mean	SD	Range	Mean	SD	Range
Block One	0.004	0.065	0–1	0.361	0.481	0–1
Block Two	0	0	0–1	0.204	0.404	0–1
**Version of the task with fillers**						
	**Monochrome-Polychrome**			**Polychrome-Monochrome**		
	**Mean**	**SD**	**Range**	**Mean**	**SD**	**Range**
Block One	0.112	0.315	0–1	0.451	0.499	0–1
Block Two	0.115	0.320	0–1	0.331	0.471	0–1

**Table 2:** Rate of RCAs for older adults on the referential communication task.

Version of the task without fillers						
	Monochrome-Polychrome			Polychrome-Monochrome		
	Mean	SD	Range	Mean	SD	Range
Block One	0.136	0.344	0–1	0.436	0.497	0–1
Block Two	0.196	0.398	0–1	0.323	0.469	0–1
**Version of the task with fillers**						
	**Monochrome-Polychrome**			**Polychrome-Monochrome**		
	**Mean**	**SD**	**Range**	**Mean**	**SD**	**Range**
Block One	0.188	0.391	0–1	0.345	0.476	0–1
Block Two	0.223	0.417	0–1	0.402	0.491	0–1

Notes: In the above tables, we divided our sample (n = 200) based on the median age such that younger adults were between the ages of 19–48 and older adults were between the ages of 50–82.

Concerning the nature of redundancy, the results are in keeping with prediction (i): we found a main effect of Order (*β* = 7.108, SE = 2.994, *z* = 2.374, *p* = 0.018) with no interaction with Age (*β* = 3.488, SE = 2.986, *z* = 1.168, *p* = 0.243). The general pattern of results revealed that irrespective of age, participants appropriately used fewer RCAs overall when presented with the monochrome to polychrome order (where there was pressure to use bare nouns in block 1, and continuing to do so in block 2 would suffice) than with the polychrome to monochrome order (where there was pressure to use RCAs in block 1 and stop doing so in block 2), supporting the view that sensitivity to efficiency pressures is maintained over the lifespan (for full model output see Supplementary Table S1).

**Figure 6:**
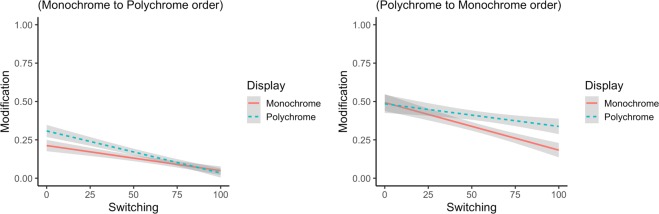
Display Type × Order × Switching interaction.

Concerning the source of redundancy, the results are in keeping with prediction (iii): we found a Display Type × Order × Switching interaction (*β* = 7.593, SE = 3.266, *z* = 2.325, *p* = 0.020) and no additional interaction with Age (*β* = 0.423, SE = 3.246, *z* = 0.130, *p* = 0.896), suggesting that the observed behaviour does not vary across the lifespan. Following up on this, we split participants by order and conducted separate analyses. Results revealed that the 3-way interaction was driven by a significant Display Type × Switching interaction only in the polychrome to monochrome order (*β* = 2.101, SE = 0.984, *z* = 2.136, *p* = 0.033), whereby adults of all ages with better switching skills appropriately used RCAs less in the second block (Fig. 6).

**Figure 7:**
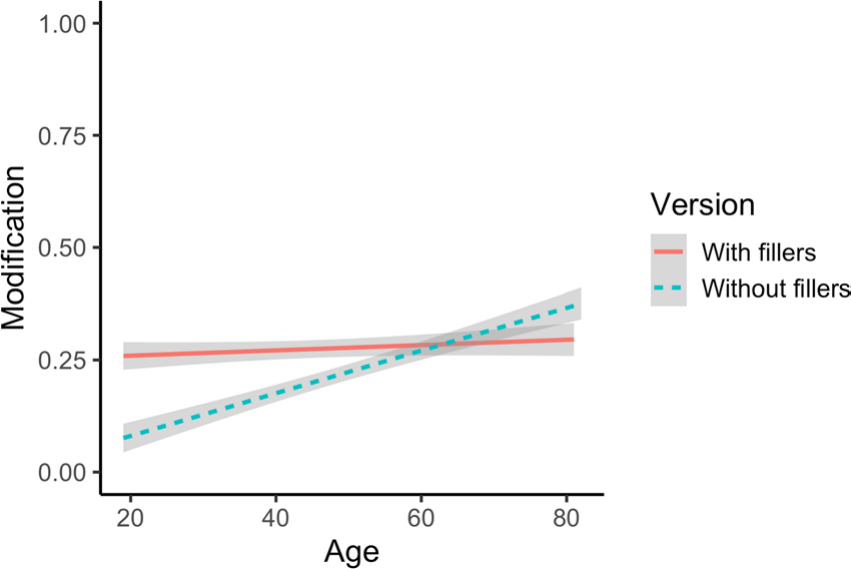
Age × Task Version interaction.

Concerning general communicative strategies, we found an Age × Task Version interaction (*β* = 6.469, SE = 2.914, *z* = 2.220, *p* = 0.026) which showed age-related differences in rate of RCAs depending on the version of the task (Fig. 7). To follow-up on this interaction, we divided participants by task version and conducted separate analyses. Results revealed that the interaction was driven by a main effect of Age only for the version without fillers (*β* = 4.588, SE = 2.142, *z* = 2.147, *p* = 0.032) such that younger adults generally modified less than older adults, perhaps because younger adults noticed that there was a higher rate of monochrome trials in the version without fillers. No other main effects or interactions reached significance.

### Additional results: rate of disfluency

**Figure 8:**
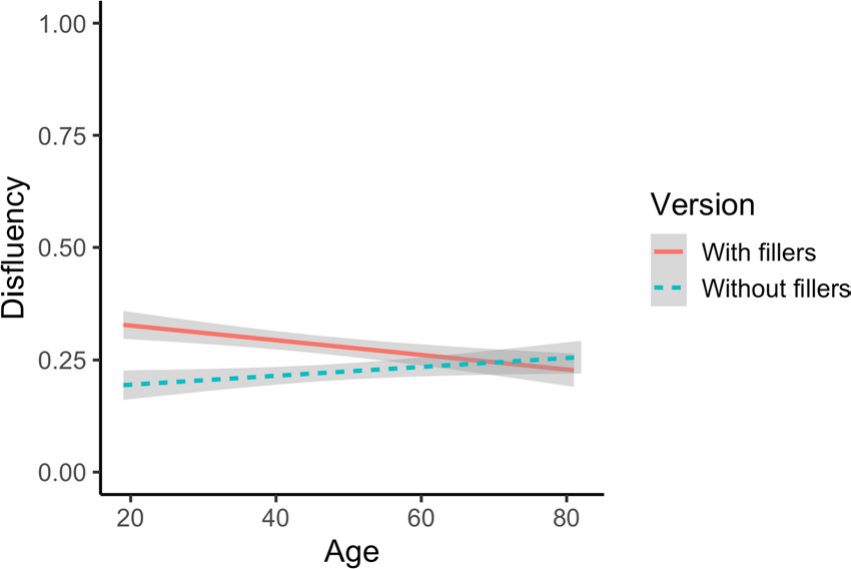
Age × Task Version interaction.

In line with our findings from the RCA analysis, our analysis of disfluency shows a main effect of Switching (*β* = −0.510, SE = 0.104, *z* = −4.893, *p* < 0.001), whereby disfluency decreased with better switching skills (for full model output see Supplementary Table S2). This supports the view that switching abilities underlie referential efficiency over the lifespan, and that fluency does not decrease with age due to a general decline in switching abilities. In addition, we found a main effect of age (*β* = −0.229, SE = 0.110, *z* = −2.070, *p* = 0.039), which revealed that younger adults were generally more disfluent than older adults. This is explained by the Age × Task Version interaction (*β* = 0.720, SE = 0.214, *z* = 3.367, *p* = 0.0008), which showed age-related differences in disfluency depending on the version of the task (Fig. 8). Follow-up analyses revealed that this interaction was driven by a main effect of Age only in the version of the task with fillers (*β* = −0.583, SE = 0.154, *z* = −3.798, *p* = 0.0001) such that as age increased disfluency decreased. This suggests that when multi-coloured displays are interspersed with monochrome and polychrome displays, younger adults’ may have had trouble deciding when to include RCAs and their production difficulty may have yielded more disfluency.

## Discussion

Here we investigated whether efficiency pressures shape referential communication across the adult lifespan. While studies from the last four decades have equated redundancy with inefficient speech, those studies have failed to account for critical perceptual factors that influence the discriminability of referents in the visual context. For example, mentioning the colour or size of an object can aid the listener’s search for the object, even if that information is redundant. In a major advance from previous studies, we tested the hypothesis that older adults’ frequent use of redundant information may not reflect insensitivity to efficiency pressures, but rather a general preference for different communicative strategies at different ages. To this end, we investigated the nature and source of age-related differences in redundancy.

To test the nature of redundancy over the lifespan (i.e. whether an increase in age results in an increase in indiscriminate redundancy or whether sensitive to context-specific efficiency pressures is maintained across adulthood) we measured participants’ use of redundant expressions in different visual contexts. Participants were shown displays in which target discriminability varied by colour (i.e. monochrome vs polychrome) in two distinct block orders where the pressure to use RCAs differed. Our results showed a main effect of order, revealing that participants appropriately used fewer RCAs in the monochrome to polychrome order (where there was less pressure to switch from using bare nouns to RCAs) than in the polychrome to monochrome order (where there was initial pressure to use RCAs). Importantly, the effect of order did not interact with participant age. These findings provide clear evidence that adults of all ages are sensitive to efficiency pressures.

To test the source of redundancy over the lifespan, we took a nuanced approach: Rather than simply attributing older adults’ use of RCAs to cognitive decline or resilience, we instead focused on the type of control strategies that are preferred by participants of different ages when responding to efficiency pressures. We hypothesized that in situations where the visual context changed, it would be especially useful to employ a reactive mode of control (through attention switching) rather than a purely proactive mode of control (through sustained inhibition), as it involves less goal-maintenance and more flexibility. Moreover, we hypothesized that a reactive mode of control may be preferred by adults of all ages, given that switching relies on a ‘late correction’ mechanism and may be less cognitively taxing than continuous sustained inhibition. Our results confirmed these hypotheses, revealing that better switching abilities were linked to more efficient linguistic behaviour that better distinguished between contexts. Importantly, this effect did not interact with participant age. Complementing these findings, we found that disfluency decreased with better switching abilities, irrespective of participant age. Together, these results challenge the notion that older adults are generally less efficient than younger adults due to age-related cognitive decline (as proposed in some studies^14–17^). Instead, our findings support the view that adults of all ages rely on a reactive mode of cognitive control for the appropriate use of facilitatory redundancy across different visual contexts.

It is worth discussing two potential counter-arguments that could be raised. One counter-argument is that because our results did not reveal a main effect of Display Type, this implies that participants were unable to distinguish between monochrome and polychrome displays. However, it is important to note that participants’ ability to distinguish between displays was likely concealed by the different efficiency pressures imposed on them by the two trial-block orders: Participants who were presented with polychrome trials in block 1 and started using RCAs should have felt pressure to stop doing so in block 2, as using RCAs in monochrome trials would be highly inefficient. However, participants who were first presented with monochrome trials should not have felt the same pressure to start using RCAs in the second block, as continuing to use bare nouns in the polychrome trials would not be comparably inefficient, given that a minimal expression does not hinder the visual search by creating unnecessary ambiguity. Indeed, in looking at the average results from block 1, participants appear to have distinguished polychrome vs monochrome trials as they consistently used more RCAs on the former trials than on the latter (see Table 1 & 2). On average, the difference between block 1 polychrome and monochrome RCA rates across ages and task versions was 0.289. This difference in polychrome and monochrome RCA rates is in line with results from previous work^10^ reporting a difference of 0.240 and a significant effect of Display Type on RCA rates when participants were presented with either monochrome or polychrome displays only. Thus, when the effect of Order is removed, the participants in our study clearly demonstrate the ability to distinguish between display types.

A second argument is that the lack of an effect of inhibition on RCA rates (revealed in our initial assessment) could be due to the fact that the TEA is not a sensitive enough test. However, Fig. 3 demonstrates that participants’ inhibition scores were not uniformly at ceiling (as was expected for the baseline measure of sustained attention). Furthermore, recent studies using the TEA have found a strong relationship between linguistic abilities and individual differences in both inhibition and switching across the lifespan^11,56^. In addition, a recent meta-analysis calls into question the widespread notion that inhibition generally declines with age^58^, in line with our results. Therefore, while we acknowledge that it is possible that the TEA was not a sensitive enough tool, we believe it is more likely that the adults in our sample were not experiencing age-related decline in inhibition, and simply preferred to rely on a reactive mode of cognitive control for facilitatory redundancy.

In addition to investigating adults’ sensitivity to efficiency pressures, we also examined age-related differences in general communicative strategies (i.e. participants’ overall rate of redundancy). We reasoned that even if adults of all ages showed sensitivity to efficiency pressures, younger and older adults might differ in the overall frequency with which they use redundant expressions. However, contrary to previous studies, we did not observe that older adults were significantly more redundant than younger adults. Nevertheless, greater redundancy in old age is visible when we look at the interaction of age and task version (i.e. the presence or absence of multi-coloured fillers). Task version can be viewed as a higher-level constraint that does not directly influence our measure of efficiency (as determined by display type and order), but may affect participants’ general communicative strategies.

In the task version without fillers, there is a clear difference between younger and older adults’ general communicative strategies (see Fig. 4): Younger adults used fewer RCAs than older adults, suggesting that they were more inclined towards a strategy of brevity. However, in the task version with fillers, younger adults displayed a similar rate of redundancy as older adults. One possibility is that younger adults noticed an increase in the proportion of multi-coloured trials in the version with fillers and responded to this increase by adopting a similar strategy as older adults: one which placed a lower premium on brevity. Supporting this interpretation is the finding that younger adults were more disfluent than older adults in the version with fillers, perhaps because younger adults noticed the subtle differences in display types and had trouble deciding whether or not to include RCAs. While these interpretations are admittedly speculative, the patterns from our data are in line with previous results showing that younger adults are highly sensitive to context: Compared to older adults, younger adults are more likely to vary the amount of descriptive information they provide depending on whether or not the information is shared with their interlocutor^32,60^. Together, these results suggest that while younger and older adults may differ in their overall rate of redundancy in the version without multi-coloured fillers, this difference does not influence their sensitivity to efficiency pressures: adults of all ages suitably reduced their use of RCAs when transitioning from polychrome to monochrome trials in this version of the task. As we hypothesized, different communicative strategies may be preferred at different ages: In the real world, a strategy which does not value brevity over redundancy may be especially well-suited for older adults as it is effective for avoiding ambiguity (i.e. underspecifying referents) and in making the signal more robust (through the use of additional attributes)^21,61^.

Overall, our findings demonstrate that efficiency continues to shape language use later in life, challenging the notion that older adults are less efficient communicators due to cognitive decline. Instead, we propose that older adults’ tendency towards redundancy may reflect a preference for different communicative strategies at different ages. Adults of all ages responded to efficiency pressures through a reactive mode of cognitive control, which is advantageous for flexible communication, as evidenced by our results and by recent work on perspective taking in referential communication^11^. Taken together, these findings have major implications for our understanding of human communication and ageing. While certain aspects of language may decline over the lifespan (e.g., word retrieval or access to phonological information^62–64^, our results demonstrate that pragmatic abilities are not affected in the same way: adults of all ages optimised their cognitive resources to maximise efficiency in referential communication. Maintaining good pragmatic skills later in life is clearly adaptive since successful communication rests on speakers and listeners coordinating efficiently. Our results therefore confirm that efficiency is a universal force that shapes communication not only across different languages, but also across the human lifespan.

## Supplementary information

Supplementary Table S1.

Dataset 1.

## Data Availability

All data generated or analysed during this study are included in this published article (and its Supplementary Information files).
