# Supplementary material for: The pressure to communicate efficiently continues to shape language use later in life
Source: Sci Rep. 2020 May 19;10:8214. doi: 10.1038/s41598-020-64475-6 (PMC7237478; doi:10.1038/s41598-020-64475-6)
Supplement: Supplementary file 1 — Supplementary Table S1. [file 41598_2020_64475_MOESM1_ESM.pdf]

The pressure to communicate efficiently continues to shape language use later in life

Madeleine Long*[1](madeleine.long@ifikk.uio.no), Hannah Rohde[2](hannah.rohde@ed.ac.uk), Paula Rubio-Fernández [1,3](prubio@mit.edu)

1. Department of Philosophy, University of Oslo, Norway, 2. Department of Linguistics, School of Philosophy, Psychology and Language Sciences, University of Edinburgh, Scotland, 3. Department of Brain and Cognitive Sciences, MIT, USA

Corresponding author's address:

Blindernveien 31
Georg Morgenstiernes hus
0313 Oslo, Norway

Supplementary Table S1.
Model output for RCA rate

Fixed effect	Coefficient	SE	P-value
Display Type	2.0103	2.0257	.3210
Order	7.1083	2.9939	.0176
Age	2.3101	1.4563	.1127
Switching	-2.2351	1.3614	.1006
Version	.9330	2.9943	.7553
Display Type x Order	3.1009	4.1398	.4538
Display Type x Age	-2.4747	1.9933	.2144
Order x Age	3.4879	2.9855	.2427
Display Type x Switching	-1.6739	1.6050	.2970
Order x Switching	-4.3160	2.7162	.1084
Age x Switching	-2.1200	1.3261	.1099
Display Type x Version	5.1838	4.0565	.2013
Order x Version	6.3367	5.9588	.2876
Age x Version	6.4692	2.9138	.0264
Switching x Version	-2.9445	2.7559	.2853
Display Type x Order x Age	-3.0273	4.0112	.4510
Display Type x Order x Switching	7.5936	3.2659	.0201
Display Type x Age x Switching	.7449	1.5891	.6393
Order x Age x Switching	-1.5204	2.6753	.5698
Display Type x Order x Version	-1.5522	8.0923	.8479
Display Type x Age x Version	2.8257	3.9690	.4765
Order x Age x Version	4.6712	5.8011	.4207
Display Type x Switching x Version	1.4716	3.2251	.6482
Order x Switching x Version	-7.3771	5.5020	.1800
Age x Switching x Version	-3.1565	2.6581	.2350
Display Type x Order x Age x Switching	.4231	3.2458	.8963
Display Type x Order x Age x Version	-7.5004	7.9708	.3467
Display Type x Order x Switching x Version	2.6918	6.4080	.6744
Display Type x Age x Switching x Version	4.1043	3.1731	.1958
Order x Age x Switching x Version	-8.4914	5.3357	.1115
Display Type x Order x Age x Switching x Version	-7.8872	6.3532	.2144

Notes: Significant main effects and interactions are shaded.

Supplementary Table S2.
Model output for disfluency rate

Fixed effect	Coefficient	SE	P-value
Display Type	-.009748	.183307	.957590
Order	.164795	.220541	.454925
Age	-.228496	.110384	.038450
Switching	-.510077	.104248	<.001
Version	-.168747	.238164	.478616
Display Type x Order	.747845	.450281	.096746
Display Type x Age	.029372	.115873	.799892
Order x Age	.093474	.213435	.661423
Display Type x Switching	.055423	.109864	.613933
Order x Switching	-.216934	.208062	.297114
Age x Switching	-.049674	.109941	.651393
Display Type x Version	-.010840	.366620	.976412
Order x Version	-.177975	.441149	.686627
Age x Version	.719840	.213811	<.001
Switching x Version	.337217	.208231	.105353
Display Type x Order x Age	-.218643	.247032	.376114
Display Type x Order x Switching	.068767	.218026	.752452
Display Type x Age x Switching	-.005344	.118046	.963891
Order x Age x Switching	.147561	.219804	.502009
Display Type x Order x Version	.940514	.735570	.201031
Display Type x Age x Version	.044638	.231713	.847239
Order x Age x Version	.409601	.426766	.337167
Display Type x Switching x Version	.153750	.217901	.480439
Order x Switching x Version	.124570	.416206	.764711
Age x Switching x Version	.125595	.226026	.578441
Display Type x Order x Age x Switching	-.262108	.236613	.267970
Display Type x Order x Age x Version	.271892	.464244	.557970
Display Type x Order x Switching x Version	.637684	.457333	.163211
Display Type x Age x Switching x Version	.097193	.236134	.680630
Order x Age x Switching x Version	.365403	.439556	.405804
Display Type x Order x Age x Switching x Version	-.449332	.472833	.341961

Notes: Significant main effects and interactions are shaded.
